# Prediction of autoimmune connective tissue disease in an at-risk cohort: prognostic value of a novel two-score system for interferon status

**DOI:** 10.1136/annrheumdis-2018-213386

**Published:** 2018-06-21

**Authors:** Md Yuzaiful Md Yusof, Antonios Psarras, Yasser M El-Sherbiny, Elizabeth M A Hensor, Katherine Dutton, Sabih Ul-Hassan, Ahmed S Zayat, Mohammad Shalbaf, Adewonuola Alase, Miriam Wittmann, Paul Emery, Edward M Vital

**Affiliations:** 1 Leeds Institute of Rheumatic and Musculoskeletal Medicine, University of Leeds, Chapel Allerton Hospital, Leeds, UK; 2 National Institute for Health Research (NIHR) Leeds Biomedical Research Centre, Leeds Teaching Hospitals NHS Trust, Leeds, UK; 3 Clinical Pathology Department, Faculty of Medicine, Mansoura University, Mansoura, Egypt

**Keywords:** autoantibodies, autoimmune diseases, cytokines, sjøgren’s syndrome, systemic lupus erythematosus

## Abstract

**Objective:**

To evaluate clinical, interferon and imaging predictors of progression from ‘At Risk’ to autoimmune connective tissue diseases (AI-CTDs).

**Methods:**

A prospective observational study was conducted in At-Risk of AI-CTD (defined as antinuclear antibody (ANA) positive; ≤1 clinical systemic lupus erythematosus (SLE) criterion; symptom duration <12 months and treatment-naïve). Bloods and skin biopsy (non-lesional) were analysed for two interferon-stimulated gene expression scores previously described (IFN-Score-A and IFN-Score-B). Forty-nine healthy controls (HCs) and 114 SLE were used as negative and positive controls. Musculoskeletal ultrasound was performed. Progression was defined by meeting classification criteria for AI-CTDs at 12 months.

**Results:**

118 individuals with 12-month follow-up were included. Of these, 19/118 (16%) progressed to AI-CTD (SLE=14, primary Sjogren’s=5). At baseline, both IFN scores differed among At-Risk, HCs and SLE groups (p<0.001) and both were elevated in At-Risk who progressed to AI-CTD at 12 months versus non-progressors, to a greater extent for IFN-Score-B (fold difference (95% CI) 3.22 (1.74 to 5.95), p<0.001) than IFN-Score-A (2.94 (1.14 to 7.54); p=0.018). Progressors did not have significantly greater baseline clinical characteristics or ultrasound findings. Fold difference between At-Risk and HCs for IFN-Score-A was markedly greater in skin than blood. In multivariable logistic regression, only family history of autoimmune rheumatic disease, OR 8.2 (95% CI 1.58 to 42.53) and IFN-Score-B, 3.79 (1.50–9.58) increased the odds of progression.

**Conclusion:**

A two-factor interferon score and family history predict progression from ANA positivity to AI-CTD. These interferon scores may allow stratification of individuals At-Risk of AI-CTD permitting early intervention for disease prevention and avoid irreversible organ damage.

## Introduction

Autoimmune connective tissue diseases (AI-CTDs) include systemic lupus erythematosus (SLE), primary Sjogren’s syndrome (pSS), systemic sclerosis, inflammatory myopathies, mixed and undifferentiated CTDs. A hallmark of their pathogenesis is loss of self-tolerance leading to autoreactivity and production of antibodies against self-nuclear antigens (ANAs). ANA can be detected in serum up to 10 years before clinical features, representing a phase of subclinical autoimmunity.^1^ However, ANA is present in up to 25% of the general population, of whom less than 1% develop clinical autoimmunity.^2 3^ Individuals with ANA therefore constitute At-Risk population of whom a minority will progress to AI-CTD.^4 5^ The factors that dictate whether this autoreactivity develops into autoimmune disease are unknown. But if these were understood and predictable, then effective intervention might be possible, preventing the severe disease and heavy glucocorticoid use for remission induction of a newly diagnosed AI-CTD.

Variants in type I interferon (IFN-I) pathway are prominent in the genetic susceptibility to AI-CTDs and therefore a focus for investigation.^6–8^ However, their role in disease initiation is currently unclear. IFN activity is usually quantified using expression of interferon-stimulated genes (ISGs). Interpretation of ISG expression is complex with multiple IFN subtypes produced by different cell types and tissues, as well as a transcriptional response in all nucleated cells with variation between cell types. Previously used IFN signatures have a categorical high/low classification^9 10^ or may have been affected by the ISGs selected.^11–13^ We recently described two continuous ISG expression scores (IFN-Score-A and IFN-Score-B) that in combination better identify clinically meaningful differences in IFN status between and within autoimmune diseases.^14^

In other autoimmune diseases such as rheumatoid arthritis (RA), early evidence of progression to disease may be found at a target tissue level.^15^ The tissues most commonly affected in AI-CTDs are the joints and skin. Musculoskeletal ultrasound can detect subclinical synovitis in SLE^16^ but has not been assessed in At-Risk individuals. In skin, specialised local immune processes are found in SLE. Previous studies comparing keratinocytes or skin biopsies isolated from patients with cutaneous lupus and healthy controls (HCs) found marked differences in IL-18R responsiveness,^17^ IFN-λ expression,^18^ as well as a role of IFN-κ in initiating a feed-forward loop, which promoted exaggerated ISG activation in cutaneous lupus.^19^ IFN-I status in the skin has not been assessed in At-Risk individuals.

The aims of this study were to evaluate clinical, blood and tissue interferon and imaging biomarkers of progression from At-Risk to AI-CTD with a view to establish a strategy for disease prevention.

## Methods

### Patients and design

A prospective observational study was undertaken in individuals who were referred from primary care to Leeds Teaching Hospitals NHS Trust due to suspected AI-CTD between November 2014 and May 2017. Inclusion criteria were (1) ANA-positive of at least 1:80 titre on indirect immunofluorescence and using multiplex immunoassays (excluding those with scleroderma (centromere, Scl-70) or myositis-specific (PL-12, OJ, PL-7, Mi-2, Ku, Jo-1, PM-Scl75, PM-Scl100, SRP and EJ) antibodies only); (2) ≤1 clinical criterion based on 2012 Systemic Lupus International Collaborating Clinics Classification Criteria (SLICC)^20^ and not meeting classification criteria for other AI-CTD^21–23^ or RA^24^; (3) symptom duration <12 months; (4) glucocorticoid, antimalarial and immunosuppressive treatment-naïve. Forty-nine HCs and 114 patients with SLE were used as negative and positive controls.

### Assessment schedule and outcome

Comprehensive assessments including clinical, laboratory, imaging, bloods and skin biomarkers were performed at baseline, 12 months and annually for 3 years. Participants were given a helpline number for an additional flare visit if they had new or worsening inflammatory symptoms. Progression was defined by meeting the 2012 SLICC criteria for SLE,^20^ 2016 ACR/EULAR criteria for pSS^21^ or other relevant classification criteria for AI-CTD^22 23^ at 12 months as assessed by rheumatologists.

### Clinical and laboratory assessment

Age, gender, ethnicity, history of first-degree or second-degree relative(s) with autoimmune rheumatic diseases (ARDs), smoking history, SLICC criteria for SLE,^20^ signs or glandular symptoms criteria for pSS,^21^ patient and physician global health assessment using 100 mm Visual Analogue Scale were recorded.

ANA was tested using indirect immunofluorescence and a panel of nuclear autoantibodies including anti-dsDNA, extractable nuclear antigens (including Ro52, Ro60, La, Sm, Chromatin, RNP, Sm/RNP and Ribosomal P) and antiphospholipid antibodies (Cardiolipin and B2-Glycoprotein IgGs) using Bioplex 2200 Immunoassay. Lupus anticoagulant tests including activated partial thromboplastin time (APTT) (Actin FS), APTT-synthetic phospholipid (with correction) and dilute Russell’s viper venom time (with correction) were deemed positive if persistent when repeated at 12 weeks. Full blood count was processed at a single accredited diagnostic laboratory. Complement levels (C3 and C4) were measured by nephelometry.

### Musculoskeletal ultrasound

Ultrasound examination of wrists, metacarpophalangeal and proximal interphalangeal joints were performed by two rheumatologists, using General Electric S7 machine with a 6–15 MHz transducer. Outcome Measures in RA Clinical Trials (OMERACT) criteria^25^ were used to define synovitis, that is, the presence of grey-scale (GS) ≥grade 2 and/or power Doppler (PD) ≥grade 1.

### Blood and skin IFN scores

A two-score system of ISGs, as previously described,^14^ was calculated without the knowledge of participant’s clinical status. See online supplementary file for details. Briefly, peripheral blood mononuclear cells (PBMCs) were separated using density gradient method (Lymphoprep; Alere Technologies, Norway) from EDTA-anticoagulated blood. Total RNA purification kit (Norgen Biotek, Canada) was used followed by quantitative real-time reverse transcriptase-PCR (qRT-PCR) using TaqMan assays (Applied Biosystems, Invitrogen) for the selected 30 ISGs.^7^ These assays were performed using the BioMark HD System with appropriate cycling protocols for the 96.96 chip. Data were normalised using Peptidylprolyl isomerase A as a reference gene to calculate ΔCt.

10.1136/annrheumdis-2018-213386.supp4 Supplementary data

Factor analysis was used to reduce the 30 ISGs into a smaller number of factors.^26^ Two factors, IFN-Score-A and IFN-Score-B, explained 84% of the variance with limited cross-loading. Factor scores were calculated as the median level of expression of the genes loaded by each factor.

### Skin biopsy

One 4 mm biopsy was obtained from non-lesional non-sun-exposed areas (upper back or upper arms) of At-Risk individuals (n=10) and HCs (n=6), and from active lesions of patients with SLE (n=10). Biopsies were snap frozen in optimum cutting temperature (OCT) compound and sectioned at a thickness of 5 µm ensuring no remaining OCT material contaminating subsequent RNA extraction/RT procedures. Gene expression analysis and calculation of factor scores were conducted as for PBMCs.

### Statistical analyses

Associations between categorical variables were tested by Fisher’s exact and Stuart-Maxwell tests for independent and paired samples, respectively. Continuous variables were compared using either Student’s t-tests or analysis of variance (ANOVA) followed by pairwise Tukey tests. For associations, Kendall’s tau-b correlation was used if ties were present, otherwise using Pearson’s correlation. Receiver operator curves (ROCs) were used to assess predictive strength and identify optimal thresholds for predicting progression to AI-CTD. For 13 At-Risk patients, gene expression data were missing at random due to samples not being processed on the day. For comparisons with HC and SLE groups, only At-Risk patients with complete data were presented. For prediction of progression, multiple imputation by chained equations was used to create 20 complete datasets, results of which were combined according to Rubin’s rules. Multivariable analyses were performed using penalised logistic regression by Lasso method.^27^ Leave-one-out cross-validation (R package cv.glmnet)^28^ identified the largest penalty coefficient lambda within 1 SE of the value that minimised deviance in each imputed dataset; average coefficients from the best models were calculated. All analyses of IFN Scores were conducted using ∆Ct scaling; results were then converted to relative expression (2^−ΔCt^) or fold difference (FD) (2^−ΔΔCt^).

Statistical analyses were performed using Stata V.13.1 (StataCorp, College Station, Texas, USA), R V.3.3.3^29^ and GraphPad Prism V.7.03 (GraphPad, La Jolla, California, USA) for Windows.

## Results

### Patient characteristics

**Figure 1:**
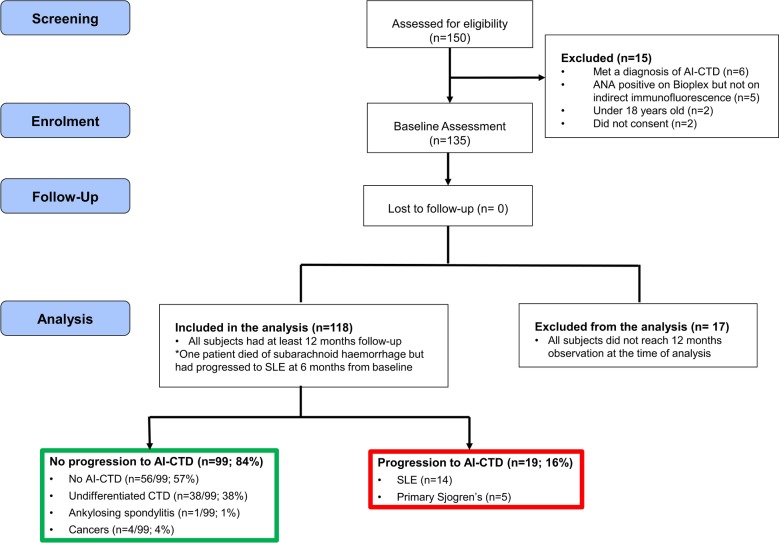
Flowchart of the At-Risk study in Leeds. AI-CTD, autoimmune-related connective tissue disease; ANA, antinuclear antibody; SLE, systemic lupus erythematosus.

The flowchart of participants is presented in figure 1. A total of 135 At-Risk individuals were recruited. Of these, 118 had at least 12 months of follow-up and were analysed. Baseline characteristics are described in table 1.

**Table 1:** Baseline characteristics of the 118 At-Risk of AI-CTD individuals

Age, median (range) years	48 (20–84)
No of female patients (%)	104 (88)
Ethnicity, N (%)	
Caucasian	85 (72)
Indian/South Asian	20 (17)
African/Caribbean	12 (10)
Chinese	1 (1)
Positive ANA, N (%)	118 (100)
No of positive ANA specificities, median (range)	1 (1–4)
Autoantibody-positive specificities, N (%)	
Anti-dsDNA	42 (36)
10–20 IU/mL	15 (13)
21–50 IU/mL	18 (15)
>50 IU/mL	9 (8)
Anti-Ro	50 (42)
<8 AI	24 (20)
≥8 AI	26 (22)
Anti-La	9 (8)
Anti-Smith	5 (4)
Anti-Chromatin	17 (14)
Anti-RNP	2 (2)
Anti-Ribosomal P	0 (0)
Anti-Sm/RNP	16 (14)
Anti-Cardiolipin/anti-B2-glycoprotein	5 (4)
Positive lupus anticoagulant, N (%)	4 (3)
Concurrent positive RF, N (%)	11 (9)
Low titre (<50 iU/mL), N (%)	5 (4)
High titre (≥50 iU/mL), N (%)	6 (5)
Concurrent positive anti-CCP antibody, N (%)*	3 (3)
Low complement levels (C3 or C4), N (%)	8 (7)
No of clinical criteria, N (%)	
0	20 (17)
1	98 (83)
Clinical criteria present, N (%)	
Acute or sub-acute cutaneous lupus erythematosus†	27 (24)
Chronic cutaneous lupus erythematosus	1 (1)
Oral or nasal ulcers	4 (3)
Non-scarring alopecia	5 (4)
Arthritis	43 (36)
Serositis	1 (1)
Renal	0 (0)
Neurological	0 (0)
Haemolytic anaemia	0 (0)
Leucopaenia or lymphopaenia	12 (10)
Thrombocytopenia	5 (4)
Glandular signs	0 (0)
Family history of autoimmune rheumatic disease, N (%)‡	43 (36)
Ever smoked, N (%)	45 (38)

*All patients had low anti-CCP antibody titre (<50 U/mL).

†Only 1 patient had SCLE lesion.

‡First-degree or second-degree relative with autoimmune rheumatic disease.

AI-CTD, autoimmune-related connective tissue disease; ANA, antinuclear antibody; CCP, cyclic citrullinated peptide; dsDNA, double-stranded DNA; RF, rheumatoid factor; RNP, ribonucleic protein.

### Clinical outcomes at 12 months

At 12 months, 19/118 (16 %) At-Risk individuals progressed to a diagnosis of AI-CTD. These were SLE (n=14; 74%) and pSS (n=5; 26%). In those who progressed, all had one clinical criterion at baseline. The number of clinical SLE criteria increased to 2 in 4/19 (21%), 3 in 9/19 (47%) and 4 in 6/19 (32%) (Stuart-Maxwell χ^2^=20.0, p<0.001) at 12 months. These details are presented in table 2 and online supplementary figure S1. Two patients developed internal organ involvement; pleural effusion and class III lupus nephritis.

10.1136/annrheumdis-2018-213386.supp1 Supplementary data

**Table 2:** Clinical characteristics of At-Risk progressors at 12 months

Clinical criteria	Baseline	12 months
	(n=19)	(n=19)
Mucocutaneous		
ACLE or SCLE	5/19 (26%)	13/19 (68%)
Mucosal ulcers	2/19 (11%)	8/19 (42%)
Alopecia	0	4/19 (21%)
Musculoskeletal		
Synovitis	9/19 (47%)	18/19 (95%)
Haematological		
Leucopaenia or lymphopenia	3/19 (16%)	7/19 (37%)
Thrombocytopenia	0	1/19 (5%)
Glandular signs	0	6/19 (32%)
Serositis		
Pleural effusion	0	1/19 (5%)
Renal		
Class III nephritis	0	1/19 (5%)

ACLE, acute cutaneous lupus erythematosus; SCLE, sub-acute cutaneous lupus erythematosus.

In contrast, 19/99 (19%) of the non-progressors had no clinical SLE criteria at both baseline and 12 months, 1/99 (1%) increased from 0 to 1, 41/99 (42%) decreased from 1 to 0 indicating a remission of autoimmunity and 38/99 (38%) had one criterion at both time points (Stuart-Maxwell χ^2^=38.1, p<0.001).

Notably, 1/99 (1%) of non-progressors had ankylosing spondylitis while 4/99 (4%) of had cancers (lung=1, hepatocellular=1, prostate=1 and leiomyosarcoma=1).

### Interferon status in At-Risk differs from SLE

**Figure 2:**
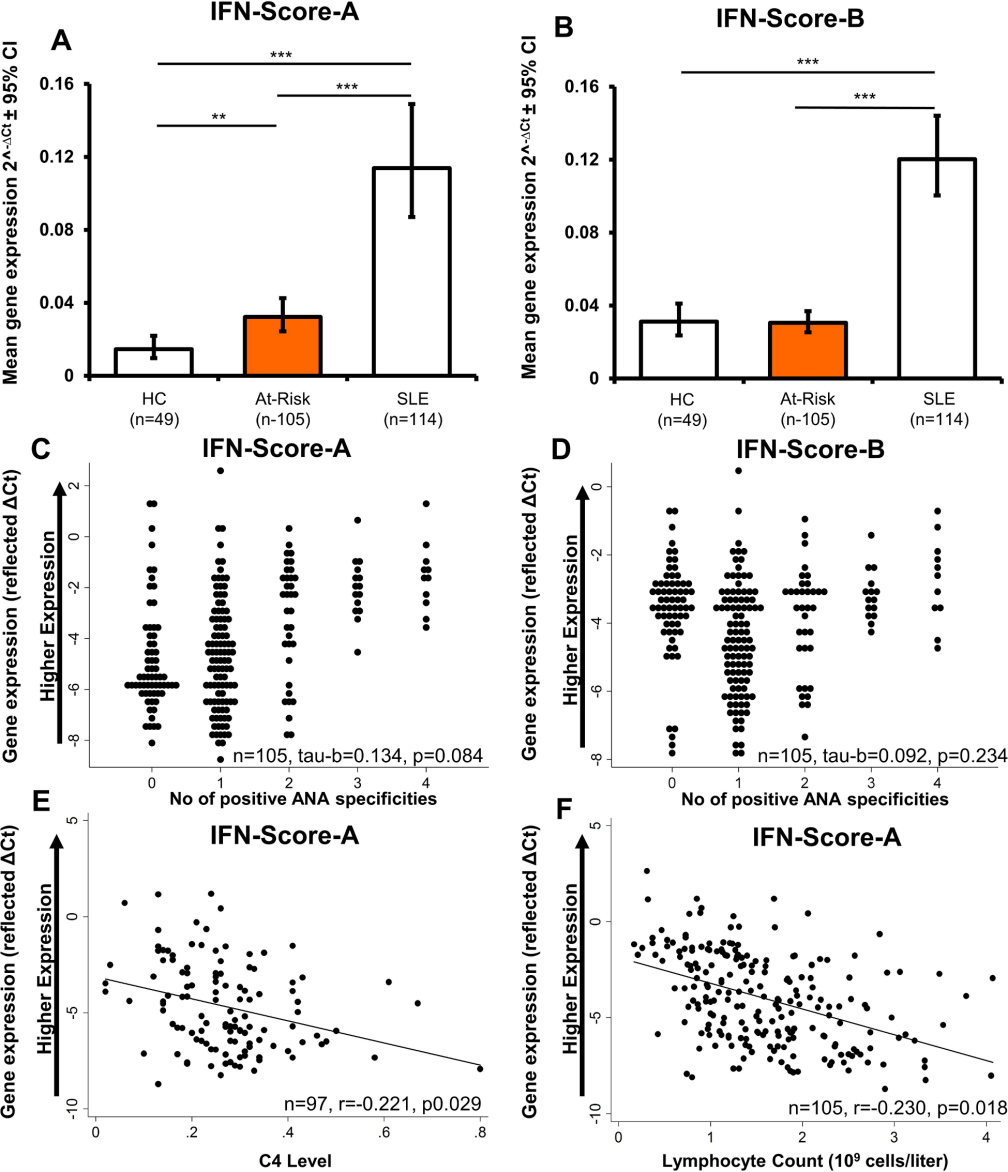
Pattern of baseline interferon scores and their relationships with clinical immunology markers. (A) Baseline expression of IFN-Score-A was higher in At-Risk individuals compared with healthy controls. (B) However, there was no difference in IFN-Score-B between both groups. ***Highly significant (p<0.001), **moderate significant (0.001<p<0.01), *significant (0.01<p<0.05). (C, D) Both IFN scores were not correlated with the number of positive antinuclear antibody (ANA) specificities (ie, sum of anti-dsDNA, Ro, La, Sm, Chromatin, RNP, Sm/RNP and Ribosomal P) and (E, F) there were only weak correlations between IFN-Score-A and complement and lymphocyte count. Data for gene expression were expressed as reflected values for ∆Ct so that higher IFN Scores represented greater expression.

At baseline, IFN-Score-A differed between groups (ANOVA F=40.26; p<0.001). It was increased relative to HC (n=49) in both At-Risk (n=105; FD (95% CI) 2.21 (1.22 to 4.00), p=0.005) and SLE (n=114; 7.81 (4.33 to 14.04), p<0.001), and was increased in SLE relative to At-Risk (3.54 (2.22 to 5.63), p<0.001) (figure 2A). In contrast, although IFN-Score-B differed between groups overall (F=63.35; p<0.001), it did not differ between At-Risk and HC (0.98 (0.66 to 1.46), p=0.993), but was increased in SLE to both HC (3.85 (2.60 to 5.72), p<0.001) and At-Risk (3.93 (2.87 to 5.37), p<0.001) (figure 2B).

### Relationships of interferon scores with autoantibodies, complement and lymphopaenia

Correlations between routine immunology markers and IFN Scores were performed in observed data using reflected ∆Ct so that higher IFN Scores represented greater expression. At baseline, there was no association between number of positive ANA specificities (ie, anti-dsDNA, Ro, RNP etc.) and IFN-Score-A (n=105, Kendall’s tau-b 0.13, p=0.084) or IFN-Score-B (tau-b 0.09, p=0.234) (figure 2C,D).

The titres of two antibodies that were mostly prevalent using Bioplex, anti-dsDNA and anti-Ro, were divided into three and two groups, respectively. There were no differences in both IFN Scores among the three anti-dsDNA groups (online supplementary figure S2A,B). Elevated levels of IFN-Score-A (FD 2.41 (95% CI 1.10 to 5.26)) but not Score-B were found in the high titre, that is, ≥8 AI anti-Ro antibody positive group (online supplementary figure S2C, D).

10.1136/annrheumdis-2018-213386.supp2 Supplementary data

There was a weak negative correlation between C4 levels and IFN-Score-A (n=97, Pearson’s r=−0.221, p=0.029) (figure 2E) but not IFN-Score-B (r=−0.089, p=0.385). There was a weak negative correlation between lymphocyte count and IFN-Score-A (n=105, r=−0.230, p=0.018) (figure 2F) but not IFN-Score-B (r=−0.127, p=0.195).

### Baseline interferon status in skin

In parallel to results obtained for PBMC, at baseline only IFN-Score-A was increased in non-lesional skin biopsies in At-Risk (n=10) versus HC (n=6); FD 28.74 (1.29 to 639.48), p=0.036. There was no difference in IFN-Score-B; FD 1.82 (0.86 to 3.86), p=0.100. As expected, both IFN Scores were higher in SLE (active lesions) compared with either At-Risk or HC; all p<0.05.

### Comparison of baseline interferon status between blood and skin

**Figure 3:**
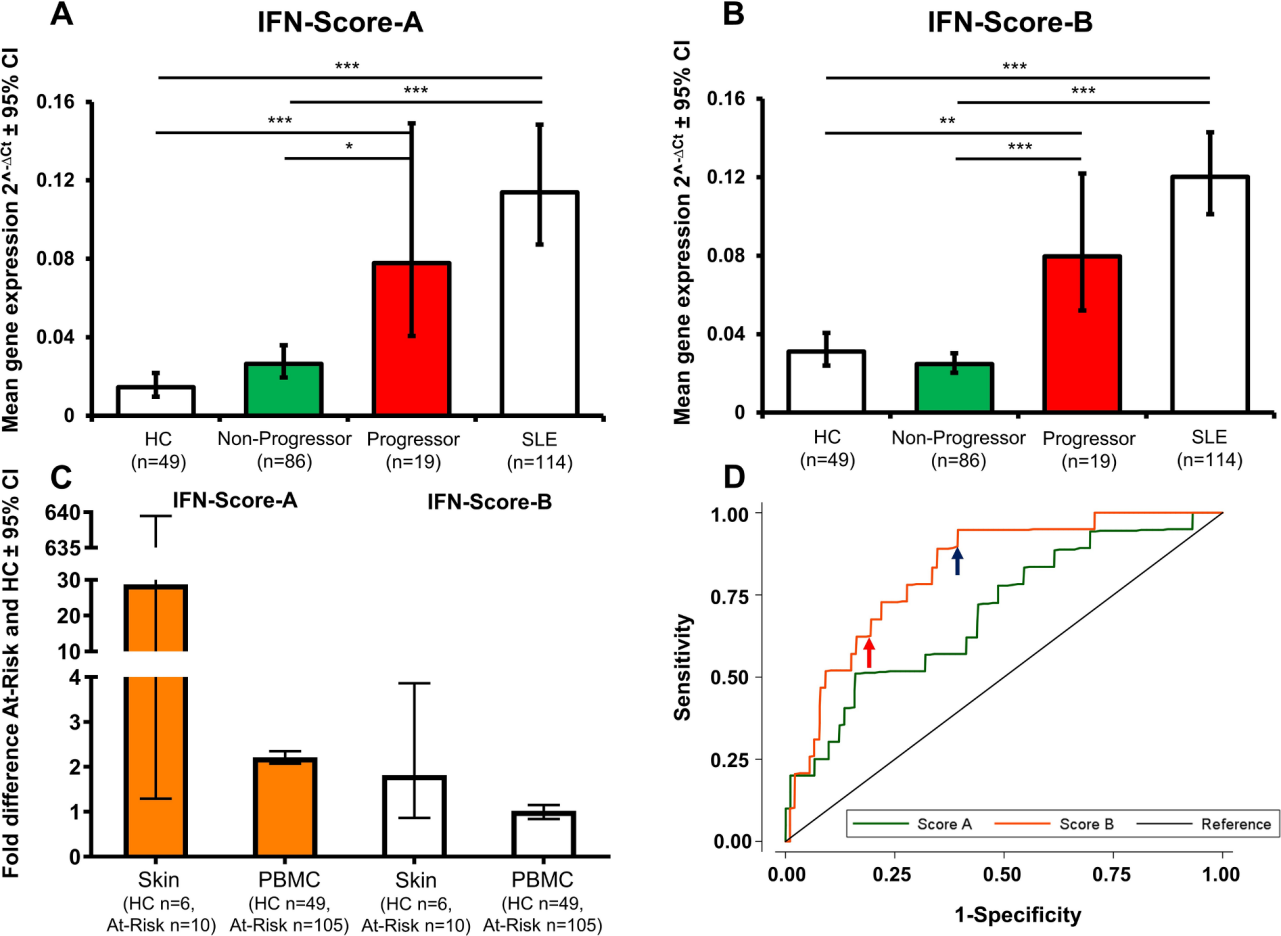
Baseline interferon (IFN) scores in bloods as prognostic biomarkers. (A–B) Baseline expression of both IFN-Score-A and IFN-Score-B were higher in At-Risk individuals who progressed to autoimmune-related connective tissue disease compared with the non-progressors, but to a greater fold difference in the latter. ***Highly significant (p<0.001), **moderately significant (0.001<p<0.01), *significant (0.01<p<0.05). (C) Fold differences for both IFN scores between At-Risk and healthy controls (HCs) were greater in skin than bloods. (D) The area under the receiver operating characteristic curve was significantly greater for IFN-Score-B than IFN-Score-A. The blue arrow denotes the optimal cut-off using Youden’s index while the red arrow denotes the proposed cut-off for prevention study. PBMC, peripheral blood mononuclear cell; SLE, systemic lupus erythematosus.

Expression of both IFN Scores was higher in At-Risk versus HC in both skin and PBMC, but FDs were greater in skin (figure 3C). This might have been due to the small sample size for skin samples (paired skin–PBMC samples were not available).

### Prediction of AI-CTD using baseline interferon scores in blood

When At-Risk were divided according to AI-CTD progression status at 12 months, both IFN Scores differed among the groups overall (p<0.001) and both were elevated in At-Risk progressors (n=19) versus non-progressors (n=86), to a greater extent for IFN-Score-B (FD 3.22 (1.74 to 5.95), p<0.001) than IFN-Score-A (2.94 (1.14, 7.54), p=0.018) (figure 3A,B). Non-progressors did not differ from HC (n=49) for both scores; IFN-Score-B (0.79 (0.51 to 1.23), p=0.520) and IFN-Score-A (1.82 (0.93 to 3.53), p=0.096). Neither IFN Score differed between At-Risk progressors and SLE (both p>0.1).

Since the number of skin biopsies obtained in At-Risk was small (n=10), no formal association between IFN Scores and progression could be determined.

### Baseline IFN-Score-B threshold of progression to AI-CTD

Prognostic ability of baseline IFN Scores to predict progression to AI-CTD at 12 months was assessed using ROC curve analysis. The area under the ROC curve was greater for IFN-Score-B (0.82 (95% CI 0.73 to 0.92)) than IFN-Score-A (0.70 (0.57 to 0.83)); χ^2^=4.19, p=0.041. A cut-off of ≤5.01 ∆Ct for IFN-Score-B maximised the Youden’s index (sensitivity+specificity−1) yielding 95% (95% CI 75% to 99%) sensitivity, 60% (50% to 70%) specificity, 35% (23% to 48%) positive predictive value (PPV) and 98% (90% to >99%) negative predictive value (NPV). However, for a rule-in biomarker for future prevention studies, a high specificity is required to exclude individuals with the lowest risk. For this purpose, we propose a cut-off of ≤3.90 ∆Ct that resulted in 68% (46% to 85%) sensitivity, 80% (70% to 88%) specificity, 43% (27% to 61%) PPV and 92% (84% to 96%) NPV (figure 3D).

### Baseline IFN Scores were lower in At-Risk without versus with one clinical criterion

All 20/118 (17%) At-Risk individuals who had no SLE clinical criterion at baseline did not progress to AI-CTD at 12 months. At baseline, FDs for both IFN scores differed among the groups overall (p<0.001) and both were lower in At-Risk with no criterion (n=17) versus with one criterion (n=88); all p<0.05 (online supplementary figure S3 in the online supplementary file).

10.1136/annrheumdis-2018-213386.supp3 Supplementary data

### Musculoskeletal ultrasound

Of the 117 At-Risk individuals with ultrasound available, 21 (18%) had ultrasound-defined synovitis at baseline (GS ≥2 only=13, PD ≥1 with or without GS ≥2=8). Of the 20 individuals who progressed, 7 (35%) had positive ultrasound at baseline versus 14% of non-progressors; p=0.050, PPV (95% CI)=33% (17% to 55%), NPV 86% (78% to 92%).

Furthermore, 43/118 of At-Risk individuals had clinical arthritis based on SLICC^20^ (8/43 (19%) had ≥2 joints with swelling or effusion while 35/43 (81%) had ≥2 joints with tenderness and early morning stiffness of ≥30 min) while 75/118 had no arthritis. In those without arthritis, ultrasound-defined synovitis was detected in 10/75 (13%) and 4/10 (40%) progressed to AI-CTD. Conversely, in those with arthritis, only 11/42 (26%) had ultrasound-defined synovitis and 3/11 (27%) progressed to AI-CTD at 12 months. Sensitivity and specificity of physician-judged arthritis with ultrasound-defined synovitis were 52% and 68%, respectively.

### Multivariable analysis of baseline predictors of progression to AI-CTD

In imputed univariable analyses, all putative predictors were associated with progression to AI-CTD at 12 months at the 10% level of significance except for complement level and lymphocyte count (both p>0.1), which were excluded from multivariable analysis (table 3). In multivariable logistic regression, family history of ARDs (OR 8.20, p=0.012) and IFN-Score-B (OR=3.79, p=0.005) were independently associated with progression. Penalised ORs remained substantive for these variables when all other variables were removed from the model. Results in complete data (n=100) were similar (data not shown).

**Table 3:** Penalised logistic regression for predictors of progression to autoimmune-related connective tissue disease at 12 months

Baseline predictors	No progression n=99	Progression n=19	Univariable OR (95% CI), p values	Multivariable OR (95% CI), p values	Penalised coefficient to OR
Age, mean (SD)	49.0 (15.8)	39.6 (11.9)	0.96 (0.93 to 0.99), 0.016	0.97 (0.92 to 1.02), 0.232	0.000 to 1.000
Ever smoked, (%)	41.8%	20.0%	0.35 (0.11 to 1.12), 0.076	0.34 (0.06 to 1.91), 0.222	0.000 to 1.000
Family history of ARDs (%)	30.6%	65.0%	4.21 (1.53 to 11.61), 0.005	**8.20 (1.58 to 42.53), 0.012**	0.243 to 1.275
No of positive ANA specificities, median (IQR)	1 (1–1)	1 (1–2)	2.07 (0.97 to 4.40), 0.060	2.41 (0.71 to 8.20), 0.161	0.000 to 1.000
Complement C4 level, mean (SD)	0.29 (0.12)	0.26 (0.08)	0.06 (0.00 to 8.05), 0.264	Excluded	Excluded
Lymphocyte count, mean (SD)	2.04 (0.77)	1.83 (0.67)	0.67 (0.34 to 1.34), 0.257	Excluded	Excluded
No of joints with positive ultrasound for synovitis, median (IQR)	0 (0–0)	0 (0–2)	1.20 (0.97 to 1.47), 0.086	1.44 (0.98 to 2.11), 0.061	0.002 to 1.002
Patient VAS, median (IQR)	36 (16–61)	47 (26–75)	1.02 (1.00 to 1.04), 0.079	1.01 (0.98 to 1.04), 0.484	0.000 to 1.000
Physician VAS, median (IQR)	11 (3–31)	31 (15–47)	1.04 (1.01 to 1.06), 0.008	1.01 (0.97 to 1.06), 0.618	0.000 to 1.000
IFN-Score-A (−ΔCt), mean (SD)*	−5.3 (1.9)	−3.8 (2.26)	1.43 (1.11 to 1.84), 0.005	0.87 (0.54 to 1.39), 0.560	0.000 to 1.000
IFN-Score-B (−ΔCt), mean (SD)*	−5.3 (1.4)	−3.7 (1.0)	2.55 (1.60 to 4.08), <0.001	**3.79 (1.50 to 9.58), 0.005**	0.319 to 1.376

*Analysis was made based on reflected ∆Ct. Thus, the higher the number, the higher the gene expression to give positive values for ORs.

ANA, antinuclear antibody; ARD, autoimmune rheumatic disease; IFN, interferon; VAS, Visual Analogue Score.

## Discussion

In this study, we report a unique cohort of At-Risk of AI-CTD individuals with longitudinal follow-up until progression to clinical autoimmunity. We demonstrate that IFN activity is strongly associated with progression independent of baseline clinical status, with measurement according to a two-score system we described being crucial. These results provide a rationale for diagnostic and preventative treatment pathways as well as assert the importance of interferons in disease initiation.

Referrals of ANA-positive individuals to rheumatologists has increased over the last decade.^30^ Concerns are that these At-Risk individuals may be discharged prematurely or be observed in an inefficient ‘watch and wait’ fashion until the diagnosis is clear, by which time the potential to prevent disease and confer the most benefit may be lost. Thus, by undertaking the largest prospective study of At-Risk individuals, which is the first to integrate clinical, imaging and immunological assessments (including skin), our findings offer a novel approach, biomarkers and have implications for future development of targeted therapies for this group of patients.

Within ANA-positive individuals, different immune phenotypes could be defined. At baseline, IFN-Score-A was elevated but not IFN-Score-B compared with HC. However, IFN-Score-B (and to a lesser degree, IFN-Score-A) were mostly elevated in those who progressed to AI-CTD. IFN-Score-A comprises many well-known ISGs that respond to IFN-I (IFN-α, IFN-β, IFN-κ, IFN-ω). In contrast, IFN-Score-B comprises ISGs that coincide with M3.4 and M5.12 modules of a previous microarray study.^7^ These ISGs were suggested to be responsive to IFN-II (IFN-γ), IFN-III (IFN-λ) as well as IFN-I. However, we cannot exclude the influence of other inflammatory mediators on this pattern of gene expression.^14^ Some studies suggested that IFN-I contributes to priming cells to secrete IFN-II.^31 32^ Conversely, a study that measured IFN activity from serum postulated a sequential role of IFN-II augmentation that led to autoantibody accumulation and subsequent elevations in IFN-α prior to SLE.^33^ Although we could not confirm which IFN pathways predominate, our findings suggest that progression to AI-CTD may not be exclusively driven by IFN-I but by a synergistic activation of ISGs induced by a range of IFNs and IFN-Score-B could act as a biomarker for more diverse immune activation.

At the tissue level, this is the first study that quantifies IFN activity in non-lesional skin of At-Risk individuals. Interestingly, similar patterns of immune dysregulation were shown between skin and PBMC. However, markedly greater FDs in both IFN scores were found in the former compared with the latter, thus highlighting skin as a potential site of AI-CTD initiation.

Only a third of the At-Risk individuals who had ultrasound-defined synovitis progressed to AI-CTD within 12 months. Additionally, small numbers of asymptomatic patients with ultrasound-detected synovitis were identified, so further work is required to determine the role of ultrasound in assessing At-Risk individuals.

Together with a family history of ARD, IFN-Score-B from blood is independently predictive of progression and is convenient as a biomarker. We have defined a cut-off level of IFN-Score-B with a moderate diagnostic accuracy in order to design a prevention study.

This study has some limitations. First, the cohort was recruited from secondary care as well as positive ANA detected by both Bioplex and indirect immunofluorescence, which might contribute to moderate-to-high pre-test probabilities for AI-CTD. Thus, our results might not be generalised to all ANA-positive cases in primary care setting. However, our cohort was quite heterogenous in terms of ethnicity and 17% of the patients had no SLE criterion at baseline. Second, we excluded individuals with scleroderma or myositis-specific only autoantibodies, which might lead to preponderance of progression to SLE or pSS. Surprisingly, one patient had a severe ankylosing spondylitis and required biological therapy. Moreover, 4% of non-progressors had cancers thus highlighting the need to be vigilant of paraneoplastic manifestation in ANA-positive individuals as well as diverse alternative diagnoses in general. Lastly, sample size was still relatively small for multivariable analysis. However, we used penalised logistic regression to minimise overfitting of data.

In conclusion, a novel ISG score, IFN-Score-B and family history of ARD predict progression from ANA positivity to AI-CTD. After validation, the predictive value of IFN scores may allow us to identify patients with imminent AI-CTD for earlier intervention using therapies that block IFNs or conventional immunosuppressants to avoid irreversible organ damage and glucocorticoid exposure. Additionally, patients with benign autoreactivity can be better identified.
