# Supplementary material for: Prediction of autoimmune connective tissue disease in an at-risk cohort: prognostic value of a novel two-score system for interferon status
Source: Ann Rheum Dis. 2018 Jun 21;77(10):1432–9. doi: 10.1136/annrheumdis-2018-213386 (PMC6161671; doi:10.1136/annrheumdis-2018-213386)
Supplement: Supplementary data [file annrheumdis-2018-213386supp004.docx]

**Md Yusof et al. Evaluating prognostic biomarkers in At-Risk cohort: A novel two-score system for interferon status predicts progression to autoimmune connective tissue disease**

**ONLINE SUPPLEMENTARY MATERIAL**

**CONTENTS:**

1. Methods: Gene probe selection and Gene expression studies
2. Table S1. List of genes analysed and probe ID
3. Methods: Factor analysis
4. Table S2: ISGs that constitute IFN-Score-A and IFN-Score-B
5. Methods: Multivariable analysis of baseline predictors of progression to AI-CTD

**METHODS**

**Gene probe selection and gene expression**

Ten genes were selected from each IFN-annotated module (M1.2, M3.4, M5.12) as reported by Chiche L et al 2014 (see Reference no.7 in the Main Manuscript), with additional common interferon-stimulated genes (ISGs) ie: IFI27 and IFI6 [total ISGs=31]. The selection of these genes were also supported by a meta-analysis of multiple GEO data biosets comparing peripheral blood mononuclear cells (PBMCs) of systemic lupus erythematosus (SLE) versus healthy control (HC) on Nextbio web engine. Peptidylprolyl isomerase A (cyclophilin A) (PPIA) was used as a reference gene (confirmed not responsive to Type 1 IFN).

**Gene expression studies**

Total RNA purification kit (Norgen Biotek, Canada) was used to extract RNA from PBMCs and sorted cell subsets. For cDNA synthesis from total RNA acquired, Fluidigm® Reverse Transcription Master Mix buffer was used according to manufacturer’s instructions including a mixture of random primers and oligo dT for priming. For skin, total RNA were extracted using RNeasy mini kit (Qiagen, Manchester, UK). The RNA quantity was measured and assessed for quality using NanoDrop spectrophotometer, ND-1000, Heathfield, UK.

TaqMan assays (Applied Biosystems, Invitrogen) were used to perform the quantitative real-time reverse transcriptase-polymerase chain reaction (qPCR) **(Table S1)**. These assays were performed using the BioMark™ HD System with appropriate cycling protocols for the 96.96 chip. Data were normalised using Peptidylprolyl isomerase A (PPIA) as a reference gene to calculate ΔCt. All analyses of IFN Scores were conducted using ∆Ct scaling; results were then converted to relative expression (2-ΔCt) or fold difference (FD) (2-ΔΔCt).

## Table S1. List of genes analysed and probe ID

| **Gene** | **ID** |  |
| --- | --- | --- |
| BST2 | Hs01561315_m1 |  |
| CASP1 | Hs00354836_m1 |  |
| CCL8 | Hs04187715_m1 |  |
| CCND2 | Hs00153380_m1 |  |
| CEACAM1 | Hs00989786_m1 |  |
| CXCL10 | Hs01124251_g1 |  |
| EIF2AK2 | Hs00169345_m1 |  |
| GBP1 | Hs00977005_m1 |  |
| GUSB | Hs99999908_m1 |  |
| HERC5 | Hs00180943_m1 |  |
| HPRT1 | Hs99999909_m1 |  |
| IFI16 | Hs00194261_m1 |  |
| IFI27 | Hs01086373_g1 |  |
| IFI44 | Hs00951349_m1 |  |
| IFI44L | Hs00915292_m1 |  |
| IFI6 | Hs00242571_m1 |  |
| IFIH1 | Hs01070332_m1 |  |
| IFIT1 | Hs01911452_s1 |  |
| IRF7 | Hs01014809_g1 |  |
| ISG15 | Hs00192713_m1 |  |
| LAMP3 | Hs00180880_m1 |  |
| MX1 | Hs00895608_m1 |  |
| NT5C3 | Hs00369454_m1 |  |
| PHF11 | Hs00211573_m1 |  |
| PPIA | Hs99999904_m1 |  |
| RSAD2 | Hs00369813_m1 |  |
| SERPING1 | Hs00163781_m1 |  |
| SOCS1 | Hs00705164_s1 |  |
| SP100 | Hs00162109_m1 |  |
| SPATS2L | Hs01016364_m1 |  |
| STAT1 | Hs01013996_m1 |  |
| TAP1 | Hs00388675_m1 |  |

**METHODS**

**Factor analysis**

Prior to factor analysis (FA), undetected ∆Ct values were singly imputed using the R package nondetects. The Kaiser-Meyer-Olkin measure was used to verify the sampling adequacy of the analysis. Principal factor extraction, without rotation, was used to identify the optimum number of factors, which was initially determined according to a parallel analysis (Monte Carlo simulation using 1000 replications). This indicated the maximum number of factors present, but if a smaller number of factors were required to explain 80% of the variance, and resulted in lower levels of cross-loading (genes loaded by 2 or more factors at >0.4), a simpler structure was selected. Having identified the number of factors present, oblique (promax; kappa=4) rotation was used to obtain the final factor solution. To calculate factor scores, within each patient, median gene expression was calculated for genes loaded at ≥0.4 by each factor, provided they did not cross-load onto more than one factor. The advantage of this approach was that it reflected the variability of the data, and respected the within-patient ordinal scaling of ∆Ct values where some genes were below the detection threshold, but yielded factor scores in units (∆Ct) that were easily interpreted in subsequent analyses.

As per our previous study (manuscript under revision), FA confirmed that ISG expression data were best described by more than one factor. A two-factor solution explained 84% of the variance with limited cross loading. There was substantive correlation between the factors in the rotated solution (r=0.56), supporting the use of oblique rotation which permitted factors to be correlated. **Table S2** shows the ISGs that contributed to each factor; we called these IFN-Score-A (comprises 12 co-clustered genes) and IFN-Score-B (comprises 16 co-clustered genes).

**Table S2: ISGs that constitute IFN-Score-A and IFN-Score-B**

| **Gene** | **Module from previous study using microarray** | **Rotated factor loading** | |
| --- | --- | --- | --- |
|  |  | **Factor 1:**  **IFN-Score-A** | **Factor 2:**  **IFN-Score-B** |
| *ISG15* | 1.2 | 0.96* |  |
| *IFI44* | 1.2 | 0.80* |  |
| *IFI27* | n/a | 0.77* |  |
| *CXCL10* | 1.2 | 0.71* | (-0.41) |
| *RSAD2* | 1.2 | 0.70* |  |
| *IFIT1* | 1.2 | 0.67* |  |
| *IFI44L* | 1.2 | 0.66* |  |
| *CCL8* | 3.4 | 0.58* |  |
| *XAF1* | 1.2 | 0.54* |  |
| *IFI6* | n/a | 0.51 | 0.45 |
| *GBP1* | 3.4 | 0.46* |  |
| *IRF7* | 3.4 | 0.46* |  |
| *CEACAM1* | 3.4 | 0.45* |  |
| *HERC5* | 1.2 | 0.43 | 0.59 |
| *EIF2AK2* | 3.4 | 0.42 | 0.64 |
| *MX1* | 1.2 | 0.40 | 0.56 |
| *LAMP3* | 1.2 |  | 0.40* |
| *IFIH1* | 3.4 |  | 0.45* |
| *PHF11* | 5.12 |  | 0.58* |
| *SERPING1* | 1.2 |  | 0.60* |
| *IFI16* | 5.12 |  | 0.64* |
| *BST2* | 5.12 |  | 0.74* |
| *SP100* | 5.12 |  | 0.74* |
| *NT5C3B* | 5.12 |  | 0.80* |
| *SOCS1* | 3.4 |  | 0.84* |
| *TRIM38* | 5.12 |  | 0.87* |
| *UNC93B1* | 5.12 |  | 0.88* |
| *UBE2L6* | 3.4 |  | 0.89* |
| *STAT1* | 3.4 |  | 0.94* |
| *TAP1* | 5.12 |  | 0.98* |
| *CASP1* | 5.12 | <0.40 | <0.40 |

**METHODS**

**Multivariable analysis of baseline predictors of progression to AI-CTD**

To assess baseline predictors of progression to AI-CTD at 12 months, initially, to account for missing data, multiple imputation by chained equations was used to create 20 complete datasets, results of which were combined according to Rubin’s rules. All (imputed) putative variables were then evaluated using univariate analysis at the 10% level of significance. Associations between categorical variables and progression to AI-CTD were tested by Fisher’s exact or chi-square while continuous variables were compared using either Student’s T-tests or Mann–Whitney test (if the data were not normally distributed). Only variables with p-value of <0.1 were included in the multivariable analysis, using backward-elimination multiple logistic regression. Multivariable analyses were performed using penalised logistic regression by Lasso method. Leave-one-out cross validation (R package cv.glmnet) identified the largest penalty coefficient lambda within 1 standard error of the value that minimised deviance in each imputed dataset; average coefficients from the best models were calculated. P-value of <0.05 was considered significant in the multivariable analysis.
