# Supplementary figures and images for: Prediction of autoimmune connective tissue disease in an at-risk cohort: prognostic value of a novel two-score system for interferon status
Source: Ann Rheum Dis. 2018 Jun 21;77(10):1432–9. doi: 10.1136/annrheumdis-2018-213386 (PMC6161671; doi:10.1136/annrheumdis-2018-213386)

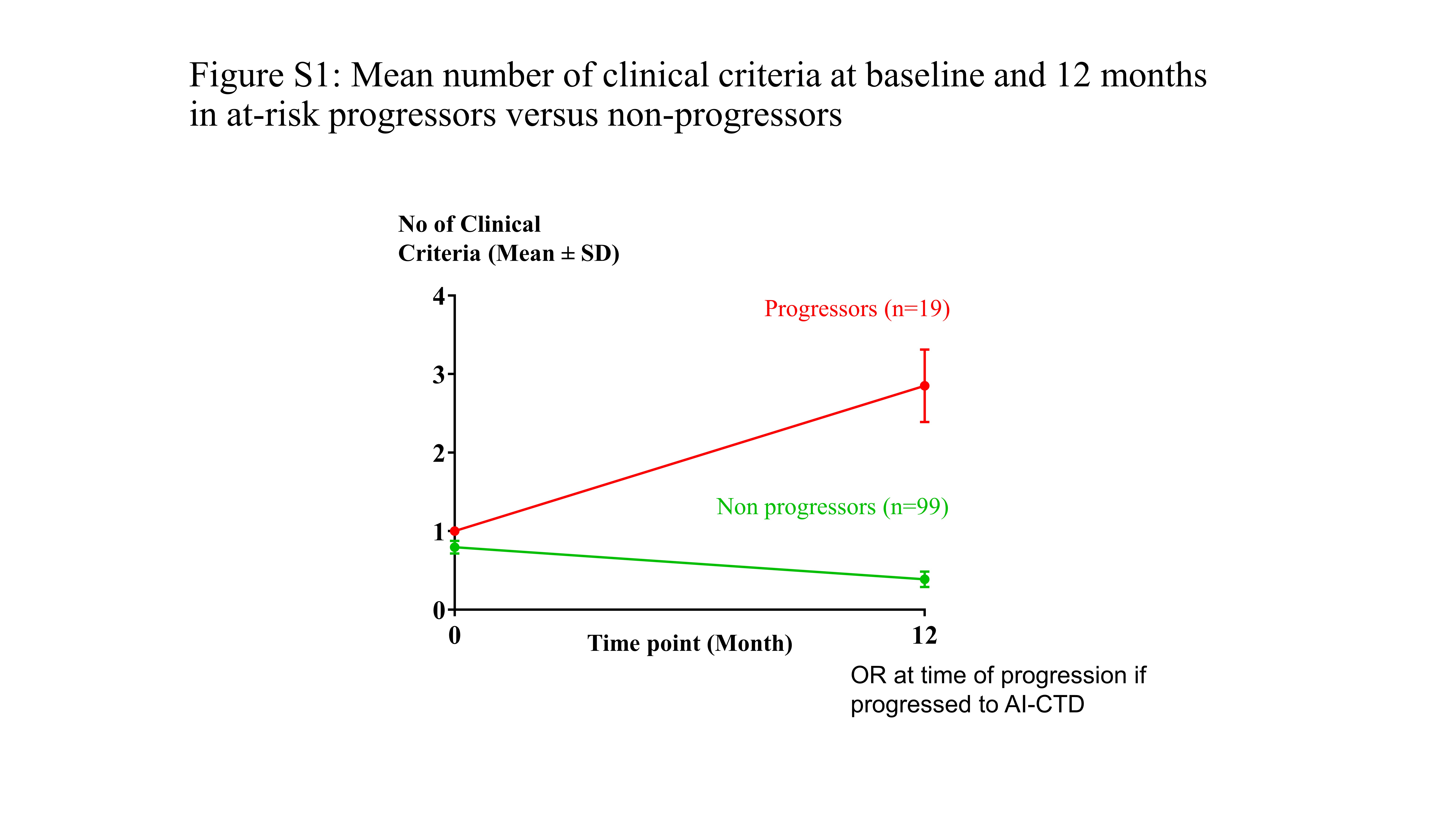

Supplement: Supplementary data [file annrheumdis-2018-213386supp001.jpg]

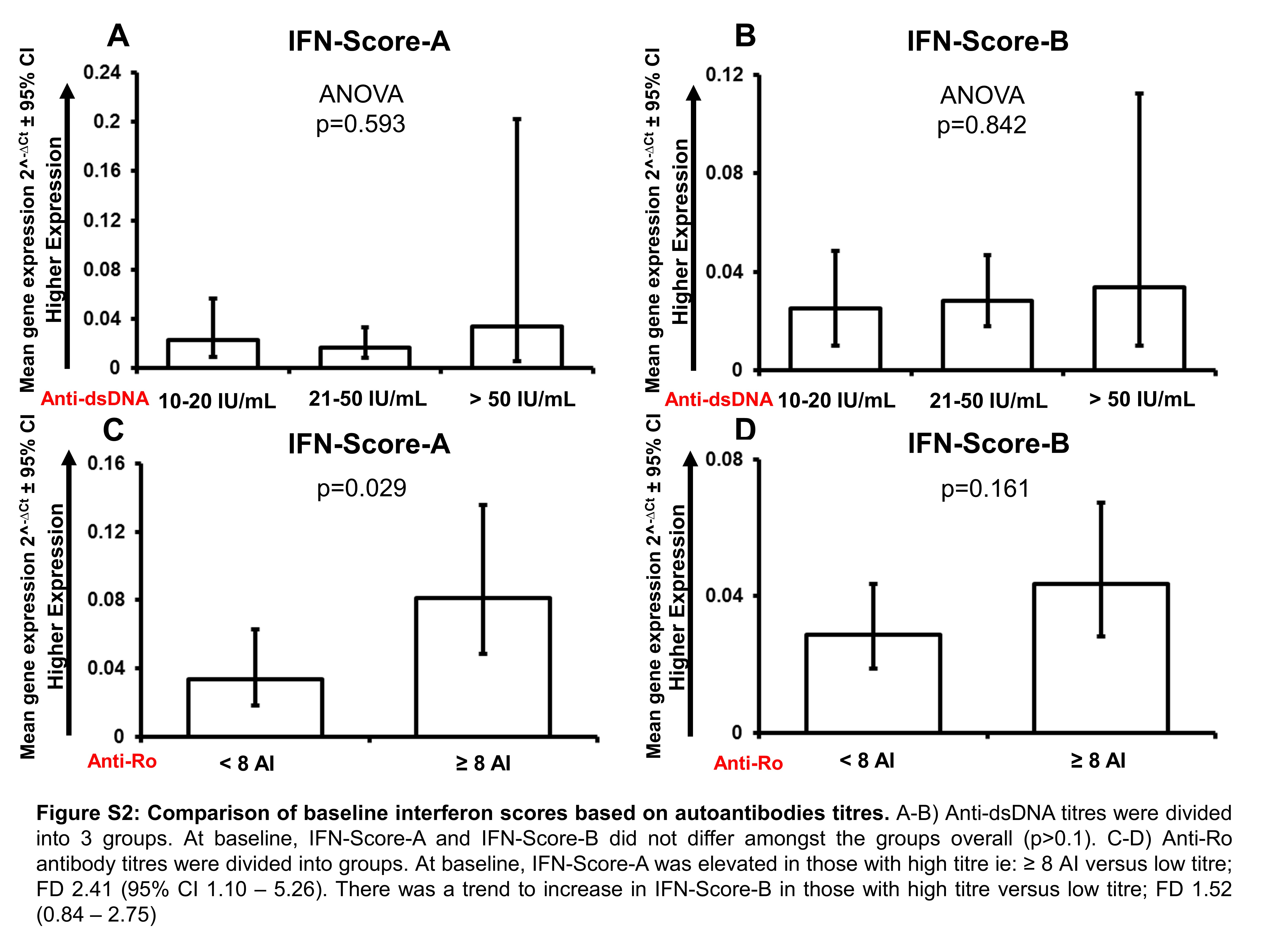

Supplement: Supplementary data [file annrheumdis-2018-213386supp002.jpg]

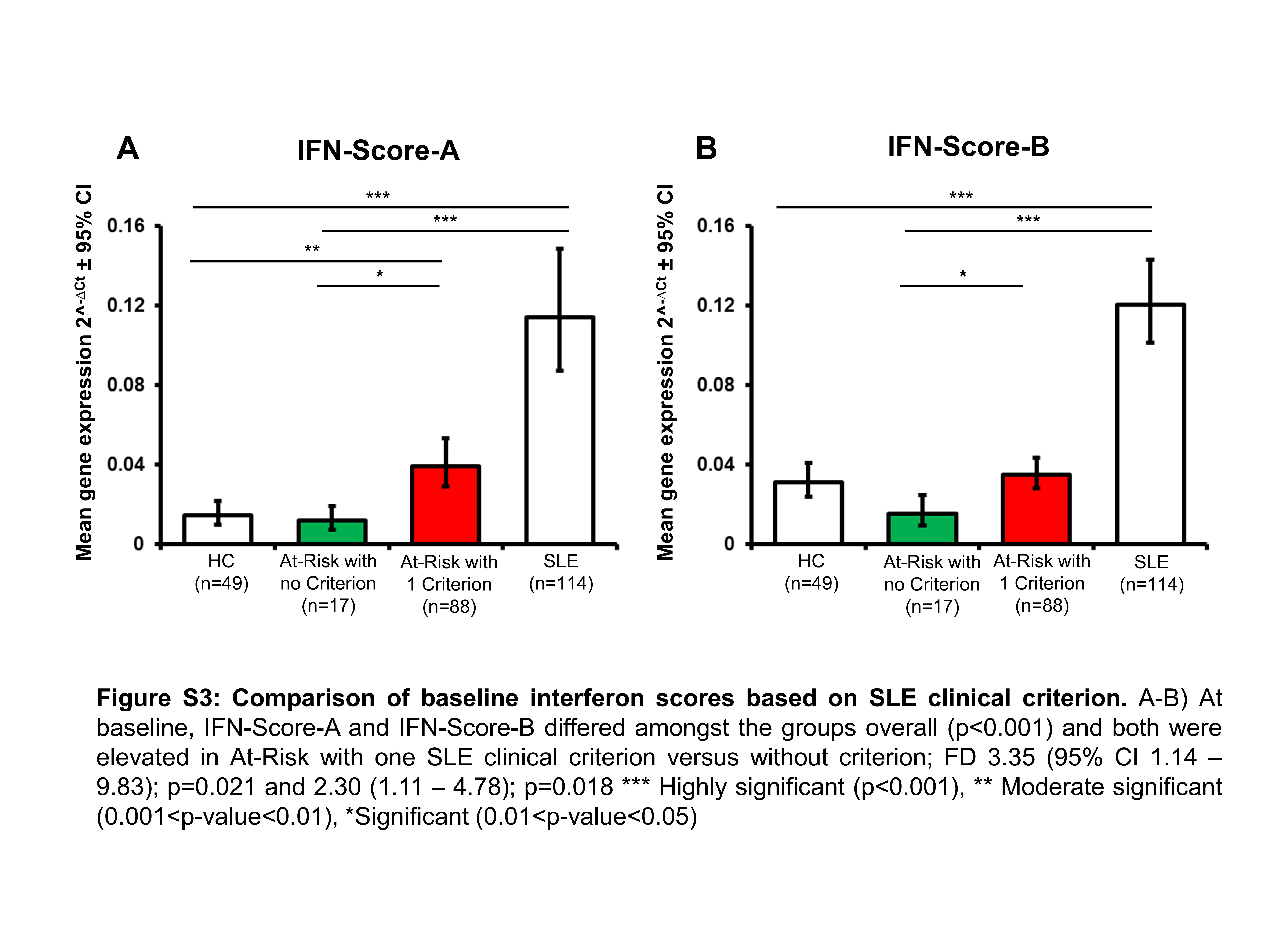

Supplement: Supplementary data [file annrheumdis-2018-213386supp003.jpg]
